# Therapie der akuten diabetischen Stoffwechselentgleisungen bei Erwachsenen (Update 2026)

**DOI:** 10.1007/s00508-025-02694-5

**Published:** 2026-04-30

**Authors:** Michael Resl, Susanne Kaser, Harald Sourij, Martin Clodi, Bruno Schneeweiß, Anton Luger

**Affiliations:** 1 Abteilung für Innere Medizin, Krankenhaus Barmherzige Brüder Linz, Linz, Österreich; 2 Klinisches Forschungsinstitut für kardiovaskuläre und metabole Erkrankungen, JKU Linz, Linz, Österreich; 3 Department für Innere Medizin 1, Medizinische Universität Innsbruck, Innsbruck, Österreich; 4 Klinische Abteilung für Endokrinologie und Diabetologie, Universitätsklinik für Innere Medizin, Medizinische Universität Graz, Graz, Österreich; 5 Intensivstation, Klinische Abteilung für Gastroenterologie und Hepatologie Universitätsklinik für Innere Medizin III, Medizinische Universität Wien, Wien, Österreich; 6 Klinische Abteilung für Endokrinologie und Stoffwechsel, Universitätsklinik für Innere Medizin III, Medizinische Universität Wien, Wien, Österreich

**Keywords:** Diabetische Ketoazidose, Hyperglykämisch-hyperosmolare Stoffwechselentgleisung, Serum-Osmolalität, Anionenlücke, Pseudohyponatriämie, Diabetic ketoacidosis, Hyperosmolar hyperglycemic state, Serum osmolality, Anion gap, Pseudohyponatremia

## Abstract

Akute Stoffwechselentgleisungen können für Erwachsene in Abhängigkeit von ihrem Ausmaß lebensbedrohlich sein. Dementsprechend sind eine rasche umfassende Diagnostik und Therapie sowie eine enge Überwachung der Vitalparameter und Laborbefunde erforderlich. Bei der Therapie, die sich bei der ketoazidotischen (DKA) und hyperglykämisch-hyperosmolaren (HHS) Form nicht wesentlich unterscheidet, kommt dem Ausgleich des meist beträchtlichen Flüssigkeitsdefizits mit mehreren Litern einer physiologischen kristalloiden Lösung eine vorrangige Rolle zu. Bei den Elektrolyten ist insbesondere auf eine ausgeglichene Serum-Kalium-Konzentration zu achten. Normal-Insulin oder rasch wirksame Analoga können initial als i.v.-Bolus verabreicht werden, in der Folge jedoch kontinuierlich über einen Perfusor. Die Umstellung auf eine subkutane Insulintherapie soll erst bei ausgeglichenem Säure-Basen-Haushalt und zufriedenstellender Glykämie erfolgen.

## Abstract (en)

Acute metabolic crises can be potentially life-threatening situations in adults depending on the extent. Therefore, rapid comprehensive diagnostic and therapeutic measures with close monitoring of vital and laboratory parameters are required. The treatment of diabetic ketoacidosis (DKA) and the hyperosmolar hyperglycemic state (HHS) is essentially the same and replacement of the mostly substantial fluid deficit with several liters of a physiological crystalloid solution is the first and most important step. Serum potassium concentrations need to be carefully monitored to guide its substitution. Regular insulin or rapid-acting insulin analogues can be initially administered as an i.v. bolus followed by continuous infusion. Insulin should be switched to subcutaneous injections only after correction of the acidosis and stable glucose concentrations within an acceptable range.

## Akute Blutzuckerentgleisungen

Ketoazidotische Stoffwechselentgleisungen (DKA) sind wesentlich häufiger als hyperglykämisch-hyperosmolare (HHS). Erstere betreffen zu zwei Dritteln Menschen mit Typ-1-Diabetes und überwiegend jüngere Patient:Innen im Alter von 18–44 Jahren [1]. Die HHS tritt im Gegensatz dazu viel häufiger bei Menschen mit Diabetes mellitus Typ 2 auf und ist auch aufgrund des höheren Alters des betroffenen Kollektivs und der damit assoziierten häufigeren Komorbiditäten mit einer deutlich höheren Mortalität verbunden (weniger als 1 % bei der DKA und 5–20 % bei der HHS; [1, 2]).

## Pathogenese, klinisches Bild und Befunde

Die Unterteilung der diabetischen Stoffwechselentgleisungen in DKA und HHS muss als grob schematisch betrachtet werden, da die DKA stets mit einer Azidose, oft aber nur mit einer mäßiggradigen Hyperglykämie verbunden ist und umgekehrt die HHS mit einem sehr hohen Blutzuckerwert, aber einer meist nur gering ausgeprägten Azidose (Tab. 1). Die DKA ist durch absolutes Fehlen von Insulin gekennzeichnet und infolgedessen fehlender Unterdrückung der Lipolyse und Verwertung freier Fettsäuren. Bei der HHS steht im Gegensatz dazu ein relativer Insulinmangel mit gesteigerter Glukoneogenese und beeinträchtigter peripherer Glukoseverwertung im Vordergrund. Im klinischen Alltag bestehen trotzdem weitgehend Überschneidungen, die HHS ist meist durch einen pH ≥ 7,3 und ein Bikarbonat von ≥ 15 mmol/l gekennzeichnet (Tab. 1). Aggravierend für die Ausbildung der Stoffwechselentgleisung sind zudem die Dehydrierung und der damit verbundene Anstieg der gegenregulatorischen Stresshormone Adrenalin, Noradrenalin und Kortisol [3–5]. Stets sollte an eine möglicherweise auslösende Ursache der Stoffwechselentgleisung gedacht werden, häufig ein Infekt, und eine ggf. erforderliche Diagnostik veranlasst werden. Weitere Ursachen für eine DKA sind häufig die Erstmanifestation eines Diabetes mellitus Typ 1, unterlassene Insulinapplikation oder Insulinpumpendefekte. Insbesondere bei der DKA kann die manchmal bestehende Pseudoperitonitis eine Herausforderung darstellen, sie muss bei der Indikationsstellung für eine Laparotomie unbedingt berücksichtigt werden. Eine Sonderform stellt die bei Therapie mit einem SGLT2(„sodium/glucose cotransporter 2“)-Inhibitor gelegentlich auftretende diabetische Ketoazidose dar, die durch eine nicht sehr stark ausgeprägte Hyperglykämie (250–300 mg/dl) gekennzeichnet ist und nicht ganz zutreffend auch als euglykämische Ketoazidose bezeichnet wird [6].

**Tab. 1:** Laborbefunde bei DKA – HHS. (Adaptiert nach [1, 2]. Aus [7])

	DKA	HHS
Plasmaglukose (mg/dl)	≥ 200	≥ 600
Arterieller pH	< 7,3	≥ 7,3
Serumbikarbonat (mmol/l)	< 18	≥ 15
Harnketone	Positiv	Negativ oder schwach positiv
Serumketone (mmol/l)	> 3,0	< 3,0
Effektive Serumosmolalität (mosmol/kg)	Variabel	> 320
Anionenlücke	> 10	Variabel
Bewusstseinslage	Klar–Koma	Stupor–Koma

Für das Auftreten einer Bewusstseinstrübung bzw. eines Komas ist vorwiegend die Hyperosmolalität verantwortlich. Bei einer Serumosmolarität < 320 mosmol/kg muss jedenfalls nach einer anderen Ursache für das Vorliegen des Komas gesucht werden, die ja auch die Ursache für die Stoffwechselentgleisung darstellen kann. Für die Beurteilung der *Serumosmolalität* ist die errechnete von größerer Bedeutung als die gemessene, wobei hier die Formel

$$\begin{aligned}\text{Serumosmolalit{\"a}t}(\mathrm{mmol}/l)=2\times \mathrm{Na}^{+}(\mathrm{mmol}/l)\\+\mathrm{K}^{+}(\mathrm{mmol}/l)+\mathrm{Glukose}(\mathrm{mg}/\mathrm{dl})\colon 18\end{aligned}$$

heranzuziehen ist, da BUN ungehindert zwischen Extra- und Intrazellulärraum diffundieren kann und in diesem Fall nicht berücksichtigt werden muss.

Die *Anionenlücke* wird anhand der Formel

$$\begin{aligned}\text{Anionenl{\"u}cke}=\mathrm{Na}^{+}(\mathrm{mmol}/l)-(\mathrm{CI}^{-}(\mathrm{mmol}/l)\\+\mathrm{HCO}_{3}^{-}(\mathrm{mmol}/l)\end{aligned}$$

errechnet [8–11] und ist bei der DKA aufgrund der Erhöhung der* K*etone ebenfalls vergrößert. Dabei ist zu berücksichtigen, dass die Anionenlücke auch bei *U*rämie, durch *S*alicylate, *M*ethylalkohol/Ethanol, *A*ldehyde, bei *L*aktatazidose und durch *E*thylenglycol vergrößert ist (Merkwort: *KUSMALE*).

Zu beachten ist ferner, dass in Abhängigkeit vom Ausmaß der Hyperglykämie auch eine *Pseudohyponatriämie* vorliegen kann: Pro 100 mg/dl über den Normbereich erhöhte Blutzuckerkonzentration ist die gemessene Serum-Natrium-Konzentration um 1,6 mmol/l niedriger [1].

## Therapie

Für die Therapie von vordringlicher Bedeutung ist zunächst der Ausgleich des Flüssigkeitsdefizits, da Insulin sonst nicht wirken kann. Durch die Hyperglykämie kommt es zu einer osmotischen Diurese, die zunächst durch eine „Autotransfusion“ von Flüssigkeit aus dem Intra- in den Extrazellulärraum ausgeglichen wird. Sie bleibt so lange bestehen, wie die Hyperglykämie besteht. Durchschnittliche Flüssigkeits- und Elektrolytdefizite bei DKA und HHS sind in Tab. 2 angeführt.

**Tab. 2:** Typische Defizite von Flüssigkeit und Elektrolyten bei DKA + HHS. (Nach [1, 2]. Aus [7])

	DKA	HHS
Flüssigkeit (l)	6	9
H_2_O (ml/kg)	100	100–200
Na (mmol/kg)	7–10	5–13
Cl (mmol/kg)	3–5	5–15
K (mmol/kg)	3–5	4–6
PO_4_ (mmol/kg)	5–7	3–7
Mg (mmol/kg)	1–2	1–2
Ca (mmol/kg)	1–2	1–2

In zahlreichen Publikationen wird eine Flüssigkeitssubstitution primär mit 0,9 % NaCl-Lösung empfohlen [1, 4, 5, 8–12]. Aufgrund der unphysiologischen Zusammensetzung (Natriumkonzentration 154 mmol/l, Chloridkonzentration 154 mmol/l) besteht aus pathophysiologischer Sicht aber das Risiko einer Verschlechterung der Azidose durch eine hyperchlorämische Azidose [13]. Besser geeignet für die Flüssigkeitssubstitution scheinen daher kristalloide Lösungen mit physiologischerer Zusammensetzung z. B. Elo-Mel® isoton (Tab. 3) zu sein. Es liegen auch Studien vor, die auf einen günstigeren klinischen Verlauf der diabetischen Ketoazidose bei Therapie mit einer kristalloiden Lösung mit einer dem Blutplasma ähnlicheren Elektrolytzusammensetzung im Vergleich zu einer Therapie mit einer 0,9 % NaCl-Lösung hinweisen [14–16]. Die Vorteile dieser Therapie-Variante werden, durch den in systematischen Reviews and Metaanalysen dokumentierten, rascheren Ausgleich der Azidose und die kürzere Dauer der Krankenhausaufenthalte belegt [17, 18]. Dies hat auch im 2024 publizierten Consensus Report, der sowohl von der European Association for the Study of Diabetes (EASD) als auch von der American Diabetes Association (ADA) unterstützt wird, seinen Niederschlag gefunden [19]. Zwar wird trotz der Evidenz für die Vorteile der Therapie mit balancierten kristalloiden Lösungen noch keine klare Empfehlung für deren bevorzugten Einsatz gegeben, diese nunmehr aber in dem Consensus Report als mit der 0,9 % NaCl-Lösung gleichwertige Flüssigkeitsersatztherapie bei diabetischen Stoffwechselentgleisungen empfohlen [19]. In der Regel sollte bei schweren Stoffwechselentgleisungen in der ersten halben Stunde bis Stunde 1 l Flüssigkeit verabreicht werden und auch in den weiteren 1,5–2 h jeweils etwa 1 l pro Stunde in Abhängigkeit von Hydratation, Diurese, Körpergewicht, kardialer und renaler Funktion sowie Elektrolyten.

**Tab. 3:** Elektrolytkonzentrationen verschiedener Infusionslösungen (im Vergleich zu Plasma). (Aus [7])

	Plasma	0,9 % NaCl	Elo-Mel® isoton	KADC	Ringer	Ringer-Laktat
Na	135–145	154	140	90	147	131
K	3,5–5,0	–	5	25	4	5,4
Ca	2,2–2,6	–	2,5	1,0	2,2	1,8
PO_4_	0,8–1,5	–	–	10	–	–
Mg	0,7–1,0	–	1,5	1,5	–	1
Cl	95–110	154	108	65	155	112
HCO_3_	21–26	–	Acetat 45	Malat 23	–	Laktat 28
Osmolalität	280–295	308	302	215	309	278

Auch auf die *Kaliumsubstitution* sollte von Anfang an größte Aufmerksamkeit gerichtet sein: Bei Kaliumkonzentrationen unter 3,5 mmol/l sollten vor Beginn einer Insulintherapie 10–20 mmol/l/h Kaliummalat (oder alternativ Kaliumchlorid) verabreicht werden, da sonst eine Aggravierung der Hypokaliämie durch den Shift von Kalium mit Insulin und Glukose von extra- nach intrazellulär und durch den Ausgleich der Azidose verursacht werden kann und die Patienten einer zusätzlichen Gefährdung ausgesetzt werden. Bei Kaliumwerten zwischen 3,5 und 5,0 mmol/l sollten 10–20 mmol Kaliummalat zu jedem Liter Flüssigkeit zugegeben werden. Lediglich bei initialen Kaliumkonzentrationen von > 5,0 mmol/l und deutlichen Hinweisen für eine Hyperkaliämie (EKG: Bradykardie, SA-Block oder höhergradiger AV-Block, hohe, spitze T‑Zacken) empfiehlt es sich, zunächst mit der Kaliumsubstitution zuzuwarten [20] und kaliumfreie Elektrolytlösungen zu verwenden.

*Insulin*, meist ein kurzwirksames Analogon, kann initial als Bolus in einer Dosierung von 0,1 IE/kgKG i.v. gegeben werden, in der Folge jedoch als kontinuierliche Infusion, die nach aktuellen Blutzuckerwerten angepasst werden muss (Tab. 4). Bei der DKA und bei der HHS soll bei Blutzuckerwerten unter 250 mg/dl und bei der euglykämen Ketoazidose bei Werten unter 200 mg/dl mit der Infusion einer 5 % Glukoselösung begonnen werden [9], um zu große und abrupte Abfälle der Glukose zu vermeiden und die weitere Insulininfusion zum Ketoazidoseausgleich ohne Hypoglykämie zu erlauben. Die Gefahr eines Hirnödems durch rasche Blutzuckerabfälle ist vorwiegend bei Kindern gegeben.

**Tab. 4:** Dosierungsschema für die kontinuierliche Insulinverabreichung mittels Perfusor. Zur Vorbereitung eines Perfusors werden 0,5 ml einer 10 ml-Durchstechflasche eines rasch wirksamen Insulinanalogs (Apidra®, Humalog®, NovoRapid®, FiAsp®, Lyumjev®; 100 IE/ml) zu 49,5 ml 0,9 % NaCl-Lösung zugegeben. 1 ml dieser Infusionslösung entspricht somit 1 IE des Insulinanalogs. (Aus [7])

Blutzuckerwerte (mg/dl)	Insulindosis
< 80	Pause, Kontrolle nach einer halben Stunde
81–120	0,7 ml/h (0,7 IE/h)
121–150	1 ml/h (1 IE/h)
151–180	1,5 ml/h (1,5 IE/h)
181–210	2 ml/h (2 IE/h)
211–240	2,5 ml/h (2,5 IE/h)
241–270	3 ml/h (3 IE/h)
271–300	3,5 ml/h (3,5 IE/h)
301–330	4 ml/h (4 IE/h)
331–360	4,5 ml/h (4,5 IE/h)
361–390	5 ml/h (5 IE/h)
391–420	5,5 ml/h (5,5 IE/h)
421–450	6 ml/h (6 IE/h)

Bei Werten über 450 mg/dl kann eine Insulindosis von 8 IE/h gewählt werden, darüberhinausgehende Dosen allenfalls in besonderen Ausnahmesituationen

N. B.: Sobald ein Patient/eine Patientin essen darf, sollten zusätzlich präprandial 1–1,5 IE Insulinanalogon/BE verabreicht werden

Für die Gabe von *Bikarbonat* besteht keine Evidenz für einen positiven Effekt, es sind auch keine Studien zu erwarten, die den Benefit einer solchen Therapie bei pH-Werten < 7 nahelegen. Es besteht aber Übereinkunft, dass bei Patienten mit einem pH-Wert < 6,9 50–100 mmol Natriumbikarbonat (50–100 ml einer 8,4 % Lösung) in 200–400 ml H_2_O über 2 h verabreicht werden sollen [1]. Allerdings sollten die zur Flüssigkeitssubstitution eingesetzten Infusionslösungen ein organisches Salz enthalten, aus welchem Bikarbonat gebildet wird.

Für die Gabe von *Phosphat* gibt es keine Evidenz hinsichtlich positiver Effekte.

Die Therapie von schweren diabetischen Stoffwechselentgleisungen sollte, wenn möglich, zunächst unter Monitoring auf einer Überwachungsstation erfolgen. Blutzuckerwerte müssen anfangs stündlich und im weiteren Verlauf 2‑ bis 3‑stündlich kontrolliert werden, um die Insulindosis anpassen zu können. Auch die Serumelektrolyte Natrium, Kalium, Chlorid und Phosphat ebenso wie Kreatinin, BUN und pH sollten anfangs in Abhängigkeit von den Ausgangswerten zunächst zumindest 2‑stündlich und dann 4‑stündlich kontrolliert werden. Da der venöse pH nur um 0,03 Einheiten niedriger als der arterielle ist, sind venöse Kontrollen des pH durchaus ausreichend, wenn keine andere Indikation für eine arterielle Blutgasanalyse gegeben ist.

Die Umstellung auf eine subkutane Insulintherapie sollte erst erfolgen, wenn die Azidose ausgeglichen ist und stabile Blutzuckerwerte in einem akzeptablen Bereich vorliegen. Der Ausgleich der Azidose beansprucht mehr Zeit als die Korrektur der Blutzuckerwerte. Zur Vermeidung eines Rückfalls der Ketoazidose sollte die Umstellung auf eine subkutane Insulingabe überlappend zur intravenösen Insulintherapie erfolgen und mindestens 2 h vor deren Beendigung eingeleitet werden.

## Interessenkonflikt

M. Resl hat von folgenden Unternehmen, die z. T. auch fördernde Mitglieder der ÖDG sind, Forschungsunterstützungen und/oder Honorare erhalten: Boehringer Ingelheim, Eli Lilly, Dexcom, Abbott. S. Kaser hat von folgenden Unternehmen, die z. T. auch fördernde Mitglieder der ÖDG sind, Forschungsunterstützungen und/oder Honorare erhalten: Boehringer Ingelheim, NovoNordisk, Eli Lilly, Sanofi, Boehringer Ingelheim, Amarin. H. Sourij hat von folgenden Unternehmen, die auch fördernde Mitglieder der ÖDG sind, Forschungsunterstützungen und/oder Honorare erhalten: Amarin, Amgen, AstraZeneca, Boehringer-Ingelheim, Bayer, Daiichi Sankyo, Eli Lilly, Novo Nordisk, Novartis, Sanofi, Sobi. M. Clodi hat von folgenden Unternehmen, die auch fördernde Mitglieder der ÖDG sind, Forschungsunterstützungen und/oder Honorare erhalten: AstraZeneca, Boehringer Ingelheim, Eli Lilly, Merck Sharp & Dohme, Novartis, Novo Nordisk, Sanofi-Aventis, Servier, Takeda. B. Schneeweiß und A. Luger geben an, dass kein Interessenkonflikt besteht.
